# Moderate rate of transmitted resistance mutations to antiretrovirals and genetic diversity in newly HIV-1 patients diagnosed in Benin

**DOI:** 10.1186/s13104-020-05151-w

**Published:** 2020-07-02

**Authors:** Edmond Tchiakpe, Rene K. Keke, Nicole Vidal, Clément Ahoussinou, Olga Sekpe, Hermione G. Dagba, Eric Gbaguidi, Conrad Tonoukouen, Aldric Afangnihoun, Moussa Bachabi, Flore A. Gangbo, Halimatou Diop-Ndiaye, Coumba Toure-Kane

**Affiliations:** 1 National Reference Laboratory of Health Program Fighting Against AIDS in Benin (LNR/PSLS), Health Ministry of Benin, BP 1258, Cotonou, Benin; 2 Laboratory of Cell Biology and Physiology, Department of Biochemistry and Cellular Biology, Faculty of Sciences and Technology (FAST) and Institute of Applied Biomedical Sciences (ISBA), University of Abomey-Calavi, 01, BP 918 Cotonou, Benin; 3 UMI233-TransVIHMI, IRD (Institut de Recherche pour le développement), U1175 (INSERM) et Université de Montpellier, Montpellier, France; 4 Epidemiologist, Socio-anthropologist, Consulting, Cotonou, Benin; 5 Health Program Fighting Against AIDS in Benin (PSLS), Health Ministry of Benin, Cotonou, Benin; 6 Centre de Traitement Ambulatoire de l’Hôpital de zone de Suru Léré, Cotonou, Benin; 7 Institute for Health Research, Epidemiological Surveillance and Training of Senegal, Dakar, Senegal

**Keywords:** HIV-1, Drug-resistance mutations, Genetic diversity, ARV naïve, Benin

## Abstract

**Objective:**

Seventeen years after the start of the IBAARV (Beninese initiative for access to antiretrovirals), transmitted drug resistance mutations in ARV-naïve patients and HIV-1 genetic diversity were investigated in Benin.

**Results:**

Drug resistance mutations were detected in (27/248; 10.9%) according to the WHO SDRM 2009 list, with a predominance of mutations directed against NNRTIs drugs (24/248; 10%). Phylogenetic and recombination analyses showed a predominance of CRF02_AG strains (165/248; 66.5%) and a high genetic diversity with five other variants and 39 URFs (15.7%) which contained portions of strains that co-circulate in Benin. Eight recent transmission chains revealed active ongoing transmission of HIV-1 strains among ARV-naïve patients. Our study showed a moderate primary drug resistance mutations rate and also provided recent data on the HIV-1 variants that circulate in Benin. Regular monitoring of primary drug resistance is required to adapt HIV-1 treatment strategies and adoption of new WHO recommendations in Benin.

## Introduction

In 2019, about 37.9 million people were living with HIV that remains a major global public health problem [1]. Seven hundred seventy thousand people died from AIDS-related illnesses and 1.7 million people became newly infected with HIV despite the expansion of antiretroviral treatment (ART) programs in 2019 [1]. An undesired consequence of antiretroviral therapy expansion is the selection of mutations [2, 3] allowing viruses to become resistant to treatment. The timing of the introduction of ART treatment programs and non-adherence can be a proxy for higher levels of pre-treatment drug resistance [4]. Transmission of these resistant viruses to therapy-naïve individuals could jeopardize the clinical benefits associated with ART [5] and the effectiveness of first-line ARV treatment. This leads to an increasing need for second-line treatment regimens whose reasons for its late start have been described by Murphy et al. [6]. Several studies reported variable rates of transmitted drug resistance ranging between 2 and 10.8% in resource-limited settings where ARTs were available [7, 8]. In West Africa, rates of 8.3% and 10.8% were reported in Niger [9] and Togo [10] while rates of 4.2% and 8.2% were reported in Morocco [11] and Cameroon [12]. The rate of 3.9% was reported in Benin in 2012 [13]. To maximize the long-term effectiveness of first-line ART, WHO recommends that HIV treatment scale-up should always be accompanied by a robust assessment of drug resistance emergence and transmission (www.who.int/hiv/topics/drugresistance/protocols/en/). One of the five keys of WHO HIV drug resistance monitoring and surveillance strategy is the surveillance of transmitted HIVDR in recently infected populations.

In Benin, HIV prevalence was 1.2% in general population in 2019. Benin reached 69-98-73 of 3X90 ONUSIDA goal and implemented a national strategy document for the care of the mother–child couple. This study reports 7 years after the first study [13] and after 17 years of ARV circulation in Benin, HIV drug-resistance mutations (DRM) and analyze whether the pattern of HIV-1 variants that circulate in Benin is stable over time in ARV-naive HIV-1 infected individuals.

## Main text

### Materials and methods

#### Study population

**Fig. 1:**
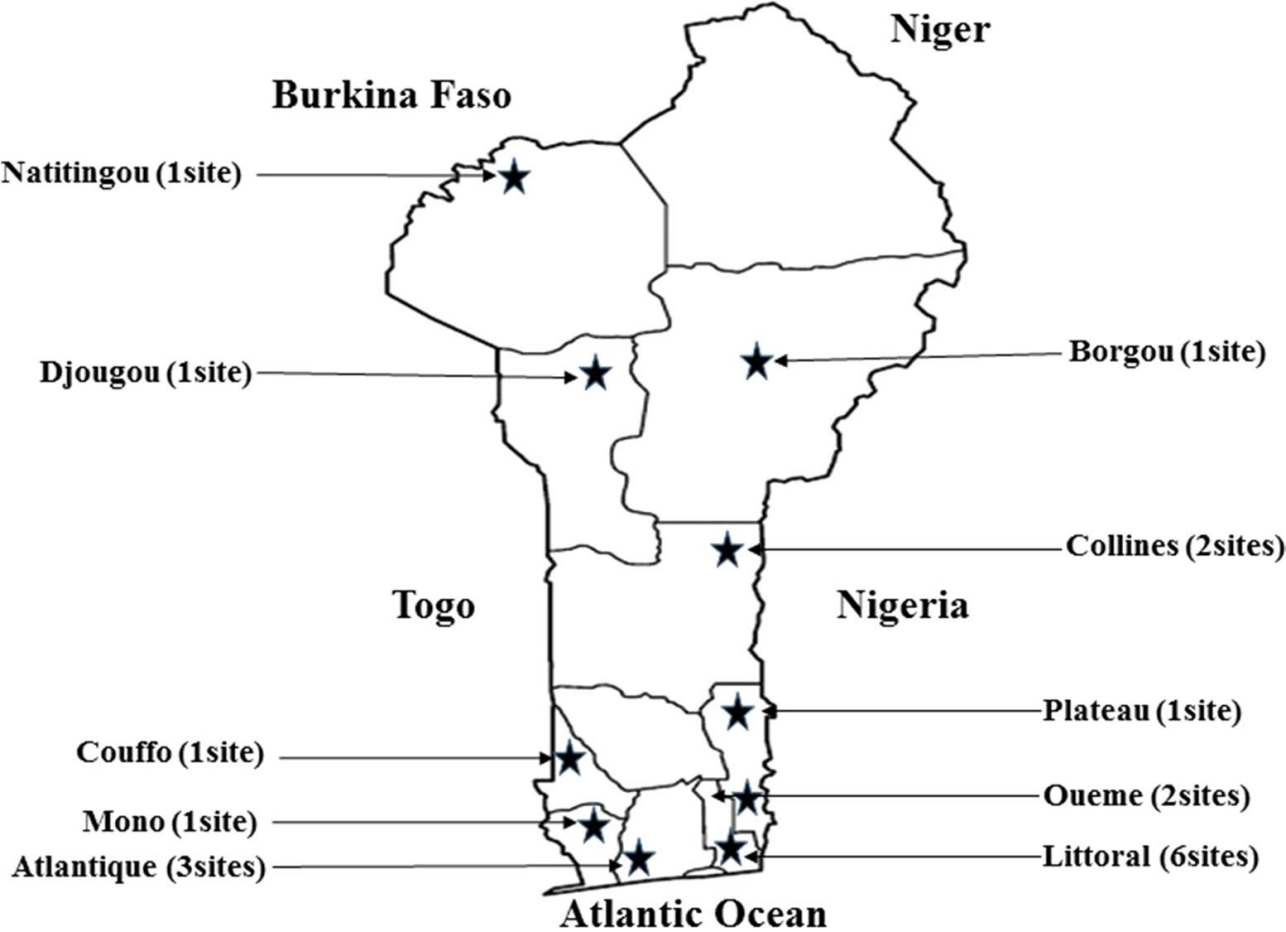
Benin geographical map showing samples collection sites. Niger, Burkina Faso, Togo, Nigeria and Atlantic Ocean are bordering of Benin. *Represent the 10 departments hosting the collection sites out of 12 in Benin

Samples (353) from antiretroviral-naïve HIV-1 infected individuals were studied. Samples were collected from October 2017 to December 2017 in nineteen health facilities care in the country (Fig. 1).

#### Blood Sampling and Processing

Blood samples were collected on EDTA tubes and RNA was extracted from plasma by using the QIAmp Viral RNA kit (Qiagen, Courtaboeuf, France) according to the manufacturer’s instructions.

#### Viral load, nested PCR amplification and sequencing

Plasma HIV-1 RNA viral load (VL) was performed using Cobas^®^ TaqMan^®^ 96/Cobas^®^ Ampliprep^®^ (CAP/CAP-CTM) HIV-1 quantitative assay (Roche Molecular Diagnostics, Basel, Switzerland) in reference national laboratory fighting against AIDS in Benin (LNR/PSLS).

Nested PCR was performed in LNR/PSLS [14] on entire protease gene and at least the first 240 amino acids encoding the reverse transcriptase as previously described [11]. After amplification testing, 248 samples were eligible for HIV-1 drug resistance genotyping

PCR products were purified (Qiagen) and sequenced on AB 3500 Genetic Analyzer using Big Dye Terminator v3.1 (Applied Biosystems, Courtaboeuf, France) after cold precipitation. Sequences were edited online (https://pssm.cfenet.ubc.ca/account/login) and translated into amino acid [15].

#### Analysis of drug-resistance mutations

Amino acid sequences were analyzed to determine the presence of mutations at positions known to be associated with drug resistance by using the latest (2009) version of the WHO list of mutations for surveillance studies [15].

#### Phylogenetic analyses

Generated sequences were aligned with reference sequences representing the overall genetic diversity of HIV-1 in West and Central Africa (available from Los Alamos HIV sequence database: http://hiv-web.lanl.gov/), by using MAFFT version 7 (https://mafft.cbrc.jp/alignment/server/) and G-Blocks to eliminate poorly aligned positions and divergent regions (molevol.cmima.csic.es/castresana/Gblocks_server). Phylogenetic tree reconstruction was done by the maximum likelihood method with the GTR + I+G model as implemented in Seaview v4.4.2 [16]. Besides, bootscanning analysis was done to explore any eventual mosaic structure for each strain with Simplot software and confirmed by phylogenetic analysis of the corresponding sub-segments in case of mosaic viruses [17].

Finally, recent transmission clusters were ascertained using the statistical robustness of ML topologies based on high bootstrap values (98%) with 1000 re-samplings and short branch lengths following criteria previously defined [18].

Newly generated sequences were submitted to Genbank database under accession numbers: MT022598 to MT022684, MT022685 to MT022845 and MT022846 to MT023006.

#### Ethics approval and consent to participate

Ethical clearance was obtained from National Ethics Committee for Health Research. Written informed consent was obtained from each study participants

### Results

#### Characteristics of 248 study patients

The median age was 38 years [IQR: 18–82] with 64.1% women and 35.9% men. Nearly 90% of patients were over 25 years of age and nearly 46% were married and living with a partner. Among patients, 4 were FSW and 8 were MSM. The median viral load was 5.12log10 [IQR: 2.20–7log10].

#### HIV DRM in antiretroviral naive patients in Benin

DRM were detected in 27 of 248 (10.9%) strains. Mutations associated with protease inhibitors (PIs), nucleoside reverse transcriptase inhibitors (NRTIs), non-nucleoside reverse transcriptase inhibitors (NNRTIs), and (NNRTIs + NRTIs) represented 1% (2/248), 6% (16/248), 10% (24/248), and 2% (5/248) respectively. Forty-two DRM have been identified (Table 1).

**Table 1:** HIV-1 Subtypes and Antiretroviral Drug Mutations in Twenty-Seven Beninese-Naive Patients

Samples ID	Age (years)	Viral load (Log_10_)	Drug Resistance Mutations Associated				HIV-1 subtypes
			PI*	NRTI*	NNRTI*	NRTI + NNRTI*	
Ben148	31	5.52	–	–	K103N	–	URF (CRF02_AG/A3)
Ben 784	34	4.31	–	–	K103N	–	CRF02_AG
Ben 538	42	5.81	–	M184V	–	–	G
Ben 169	33	6.2	–	–	G190A	–	URF (CRF06_cpx/A)
Ben 18	36	5.04	I85V	–	–	–	CRF02_AG
Ben 226	35	5.7	–	–	K103N	–	A3
Ben 230	25	5.37	–	–	K103N	–	CRF02_AG
Ben 232	20	6.57	–	M184V	–	–	URF (CRF02_AG/A1)
Ben 255	30	5.39	–	–	K103N	–	CRF02_AG
Ben 722	29	5.28	–	D67N	K103N	D67N, K103N	CRF02_AG
Ben 607	40	3.45	–	M184V	V106A, G190A	M184V, V106A, G190A	G
Ben 96	31	5.85	L90M	–	–	–	CRF02_AG
Ben 98	43	4.97	–	–	K103N	–	CRF06_cpx
Ben 687	28	5.28	–	M41L	K103N	M41L, K103N	URF (CRF06_cpx/CRF02_AG)
Ben 420	25	631	–	M184V	–	–	URF (CRF06_cpx/CRF02_AG/A3)
Ben 643	20	5.54	–	–	Y188L	–	CRF02_AG
Ben 657	42	5.6	–	T215S	–	–	CRF02_AG
Ben 206	41	5.27	–	–	K103N, P225H	–	CRF02_AG
Ben 208	26	4.99	–	M184V	–	–	CRF02_AG
Ben 211	51	4.31	–	–	K103N	–	CRF02_AG
Ben 212	35	4.18	–	K65R, M184I	K103N, Y188L	K65R, M184I, K103N, Y188L	CRF02_AG
Ben 213	35	5.58	–	M184V	Y181C	M184V, Y181C	G
Ben 214	45	5.97	–	–	K103N	–	CRF02_AG
Ben 216	37	6.68	–	M184V	–	–	URF (CRF02_AG/U)
Ben 218	52	5.06	–	–	K103N	–	A3
Ben 390	52	5.67	–	–	K103N	–	CRF02_AG
Ben 101	45	5.39	–	D67G, K70R, M184V K219Q	V106M, Y181C, G190A	D67G, K70R, M184V, K219Q V106M, Y181C, G190A	G

*NRTI* nucleoside reverse transcriptase inhibitors,* NNRTI* nonnucleoside reverse transcriptase inhibitors,* PI* protease inhibitors,* URF* unique recombinant forms,* Ben* Benin

*According to the WHO algorithm for Surveillance of Drug Resistance Mutations (SDRM), 2009

Two patients harbored each one protease mutation (I85V, n = 1) and (L90M, n = 1).

The RT gene revealed mutations selected by thymidine analogs. One patient was infected with HIV-1 containing K65R and 19% of naïve patients carried M184V (n = 8).

NNRTI and (NRTI + NNRTI) resistance mutations were detailed in Table 1.

#### The distribution of 248 HIV-1 group M variants in Benin

**Fig. 2:**
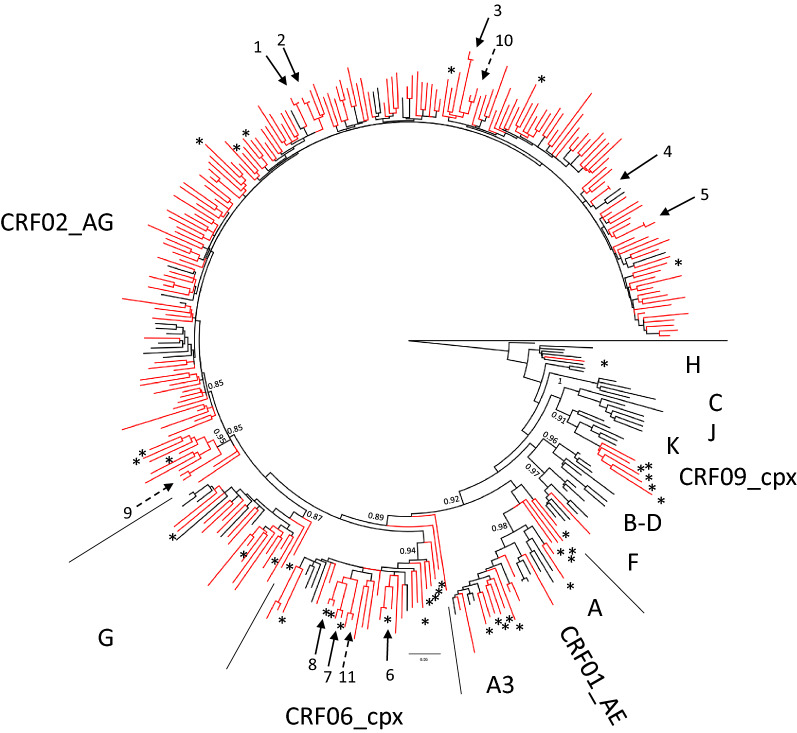
Maximum likelihood tree for 248 HIV-1-infected drug-naïve patients in Benin. The length of the alignment was 656 unambiguously aligned nucleotides in the reverse transcriptase region of the *pol* gene. Circulating recombinant forms (CRFs) and sub-subtypes not represented within the sequences genomic structure were excluded. Reference sequences are in black and patient sequences are in red. Unique recombinants over protease and RT are shown with an asterisk. An arrow indicates the sample sequences involved into recent transmission chains (n = 8), older supposed transmission chains are indicated with a broken arrow (n = 3), and the clusters were numbered. Branch support values are indicated for the major key nodes

The phylogenetic tree analysis of 248 pol sequences is presented in Fig. 2. CRF02_AG predominated and represented [165/248 (66.5%)] of the strains, followed in decreased order by CRF06_cpx [19/248 (7.7%)], subtype G [16/248 (6.5%)], sub-subtype A3 [6/248 (2.4%)], A [1/248 (0.4%)], F [1/248 (0.4%)]. Moreover, 39/248 (15.7%) strains were unique recombinants (URFs), composed with strains that predominate or co-circulate in Benin: CRF02_AG, CRF06_cpx, G and A3.

#### Identification of transmission clusters

A maximum-likelihood phylogeny with 1000 bootstraps was inferred among thirty sample sequences suspected to share links (Additional file 1: Figure S1). Phylogenetic analysis evidenced eight recent transmission chains based on high bootstrap values (98%) with 1000 re-samplings and very short branch lengths (< 0.015). Three other older transmission clusters were supposed with branch lengths values 0.016 and 0.018 (Additional file 1: Table S1).

## Discussion

The study reports the presence of mutations in ARV-naive patients and genetic diversity of HIV-1 variants that circulate in Benin. Globally, the prevalence of transmitted drug resistance was 10.89% after 17 years of ARV circulation in the country, that is consistent with results from other African countries [10, 19–21]. But, studies from some other Sub-Saharan countries have reported rates lower than 10%, by using the same WHO standard protocol and the WHO list of resistance mutations for epidemiological surveys [22, 23]. HIV-1 drug resistance mutations were known to be one of the major factors limiting the effectiveness of ARVs. In our study, the mutations encountered were those associated with the ARVs used in first-line treatment since the start of IBAARV in 2002.

This first-line treatment used two NRTIs (zidovudine/stavudine (AZT/D4T) + lamivudine (3TC)) plus an NNRTI [nevirapine/efavirenz (NVP/EFV)], as well as non-boosted protease inhibitor (indinavir) regimens [24]. At that time, virologic monitoring was not available and patients were followed based on clinical signs and CD4 counts [25]. Moreover, those who were in therapeutic failure stayed long periods with ineffective treatment, leading to an accumulation of resistance mutations [26]. This accumulation of resistance mutations may compromise the effectiveness of second-line drugs [27] and increases the risk of transmission of drug-resistant strains to naïve patients [10]. Among naïve patients in our study, 27 already harbored at least one drug resistance mutations and the NNRTIs represent 10% while the NRTIs and PIs represent 6% and 1% respectively.

NRTIs resistance-associated mutations were present in sixteen patients. M184V was the predominant NRTI encountered which confers resistance to 3TC. The K65R confers resistance to abacavir (ABC) and tenofovir (TDF). Thymidine-associated mutations (TAMs) were found but only one patient in our study harbored at least two TAMs conferring intermediate resistance to zidovudine (AZT). TAMs M41L, T215S have been described in Togo [10, 21] and also in Burkina Faso with D67N and K219Q [21]. K70R observed in one case in our study was also found in Guinea-Conakry [23]. For non-adherence reasons to treatment, M184IV is quickly selected in patients under 3TC which explains its presence in high proportion in studies of transmitted resistance [22, 23]. The high predominance of M184V could also mean that these individuals are not naive due to the infrequent transmission of this mutation, which is due to its high fitness. Lamivudine is also given to HBV infected individuals or individuals co-infected with HIV and HBV. Since the study was conducted after the time of TDF use (replacing D4T as recommended by WHO), the presence of K65R in patients could easily be explained.

The major mutations associated to NNRTIs were K103N (33%) encountered in fourteen patients which compromise the effectiveness of NNRTIs first generation (NVP and EFV) and G190A (7%) identified in three patients which compromise NVP, EFV and etravirine [28]. The high prevalence of the K103N mutation could be explained by its transmission capacity similar to that of wild-type viruses and may be present for years in infected individuals [29–31]. This mutation is also the most common in women who receive a single dose of NVP for the prevention of mother-to-child transmission [32, 33]. In fact, approximately 64.1% of study participants were women and some of them could be part of PMTCT programs. These mutations associated with high-level resistance have been described in one and four patients in Togo and Conakry [10, 23] respectively in naïve patients. The others mutations excepting Y181C and Y188L detected in two cases were V106A, P225H, and V106M, each detected in one case.

Two people exhibited each one mutation associated to PIs (I85V, n = 1) and (L90M, n = 1). In our study, no patient harboured both mutations which conferred intermediate resistance to atazanavir.

Globally, the presence of these mutations could be explained by the wide use of Triomune at the start of IBAARV in Benin [24].

Phylogenetic analysis evidenced eight recent and three probable older transmission chains (6.5–8.9% of the study patients), reflecting active ongoing transmission. Interestingly, eight patients reported as being MSM and from them, two were involved into the same transmission chain, in which one patient came from Togo and the second one was from Benin. This situation was described in Dakar where the subtype C predominating in the MSM group is increasing in the general population [34, 35], confirming the existence of a dual epidemic in the country. In our study we did not find any transmission chain involving both population groups individual, however, the number of MSM patients is too low to conclude. Further studies are needed in key groups to assess whether HIV-1 strains from MSM intermix with those from the general population in Benin or with those from other countries.

CRF02_AG predominated with 66.5% rate confirming the stability of genetic diversity in 2011 [24] and 2012 [13]. Overall, the genetic diversity in Benin matches with results found in a neighboring country, Togo [10]. The other strains (CRF06_cpx, subtype G and sub-subtype A3) have been also reported in other neighbouring African countries [9, 22, 36]. Regarding the unique recombinants (URFs), their proportion among different regions have changed over time [37]. The rate observed in our study (15.7%, 39/248) is fully concordant with the proportion from West Africa [37] and is not significantly different from that was observed in Togo (22.9%, 19/83) (p = 0.27). These URFs being composed of predominant or co-circulating strains, demonstrate the existence of frequent dual infections with at least two strains of HIV-1 [38] in the country.

The study documents the moderate prevalence of transmitted drug resistance of HIV-1 in Benin, and active ongoing transmission of HIV-1 strains circulating usually in West Africa.

## Limitations

Our study has certain limits. Patients who reported being naive may have been on ART and have previously harbored resistant viruses because they were included based on self-reported information. Moreover, the number of patients recruited is higher in the south of country than the north.

## Supplementary information

**Additional file 1: Figure S1.** Phylogenetic analysis of transmission chains. Maximum likelihood tree with 1000 bootstrap resamplings for 30 sample sequences (in red). Bootstrap values are indicated on the branches and clusters are numbered. **Table S1.** Branch lengths for each cluster or sequences pair indicated in the tree of Figure S1.

## Data Availability

All the raw data generated are available upon reasonable request to corresponding author

## Abbreviations

IBAARV: Beninese initiative for access to antiretrovirals
CNERS: National Ethics Committee for Health Research
ARV: Antiretroviral
Pol: Polymerase
RT: Reverse transcriptase
WHO: World Health Organization
Pb: Base pairs
CD4: Cluster of differentiation 4
DRM: Drug-resistance mutations
SDRM: Surveillance Drug Resistance Mutation
URF: Unique recombinant form
CRF: Circulating Recombinant forms
ART: Antiretroviral Treatment
HIV: Human Immunodeficiency Viruse
HIVDR: Human Immunodeficiency Viruse Drug Resistance
EDTA: Ethylenediaminetetraacetic
RNA: Ribonucleic acid
TAMs: Thymidine-associated mutations
VL: Viral Load
ANRS: National Agency for Research on AIDS and Viral Hepatitis
MAFFT: Multiple sequence alignment based on fast Fourier transform
PCR: Polymerase Chain Reaction
IQR: Interquartile
NNRTI: Non-nucleoside reverse transcriptase inhibitor
NRTI: Nucleoside reverse transcriptase inhibitor
PI: Protease inhibitor
MSM: Men having sex with Men
FSW: Female sex worker
AZT: Zidovudine
SQV: Saquinavir
IDV: Indinavir
NFV: Nelfinavir
FSP: Fosamprenavir
TPV: Tripanavir
LPV: lopinavir
ATV: atazanavir
RPV: Rilpivirine
DOR: Doravirine
ABC: Abacavir
TDF: Tenofovir
EFV: Efavirenz
NVP: Nevirapine
ETR: Etravirine
A: Alanine
C: Cysteine
D: Acide aspartique
E: Acide glutamique
F: Phenylalanine
G: Glycine
H: Histidine
I: Isoleucine
K: Lysine
L: Leucine
M: Methionine
N: Asparagine
P: Proline
Q: Glutamine
R: Arginine
S: Serine
T: Threonine
V: Valine
W: Tryptophane
Y: Tyrosine
