# Supplementary material for: Moderate rate of transmitted resistance mutations to antiretrovirals and genetic diversity in newly HIV-1 patients diagnosed in Benin
Source: BMC Res Notes. 2020 Jul 2;13:314. doi: 10.1186/s13104-020-05151-w (PMC7330984; doi:10.1186/s13104-020-05151-w)

Table S1 : Branch lengths for each cluster or sequences pair of the tree.

| Cluster /Pair | Branch length isolate 1 | Branch length isolate 2 | Average |
| --- | --- | --- | --- |
| 1 | 0.0152664 | 1.21e-06 | 0.008 |
| 2 | 0.0012134 | 0.0113204 | 0.006 |
| 3 | 1.9e-07 | 0.0139453 | 0.007 |
| 4 | 0.00000006 | 0.00000002 | 0 |
| 5 | 0.0183187 | 0.00000002 | 0.009 |
| 6 | 0.0000008 | 0.02311 | 0.011 |
| 7 | 0.0165417 | 0.0135496 | 0.015 |
| 8 | 0.0148238 | 0.00593558 | 0.010 |
| 9 | 0.0204164 | 0.0124751 | 0.016 |
| 10 | 0.00933966 | 0.0269886 | 0.018 |
| 11 | 0.0117215 | 0.024674 | 0.018 |
| Pair A | 0.0244914 | 0.0406802 | 0.032 |
| Pair B | 0.0126466 | 0.0314102 | 0.022 |
| Pair C | 0.0229543 | 0.0393063 | 0.031 |
| Pair D | 0.0143373 | 0.0340017 | 0.024 |


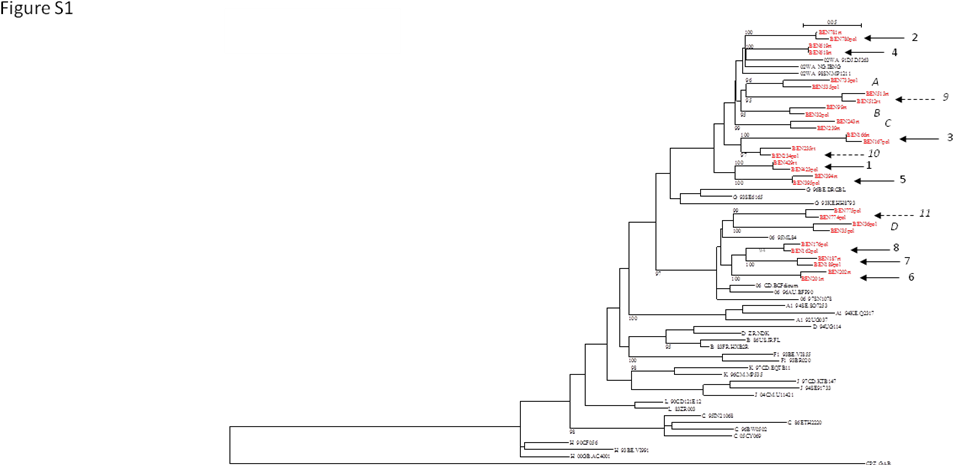

Supplement: Supplementary file 1 — Additional file 1: Figure S1. Phylogenetic analysis of transmission chains. Maximum likelihood tree with 1000 bootstrap resamplings for 30 sample sequences (in red). Bootstrap values are indicated on the branches and clusters are numbered. Table S1. Branch lengths for each cluster or sequences pair indicated in the tree of Figure S1. [file 13104_2020_5151_MOESM1_ESM.doc]
